# Does Mandibular Gonial Angle Influence the Eruption Pattern of the Lower Third Molar? A Three-Dimensional Study

**DOI:** 10.3390/jcm10184057

**Published:** 2021-09-08

**Authors:** Selene Barone, Alessandro Antonelli, Fiorella Averta, Federica Diodati, Danila Muraca, Francesco Bennardo, Amerigo Giudice

**Affiliations:** 1 Department of Health Sciences, School of Dentistry, Magna Graecia University of Catanzaro, 88100 Catanzaro, Italy; barone.selene19@gmail.com (S.B.); antonellicz@gmail.com (A.A.); fiore.averta@gmail.com (F.A.); fefediodati@gmail.com (F.D.); muracadanila@gmail.com (D.M.); fbennardo92@gmail.com (F.B.); 2 Department of Health Sciences, Maxillofacial Surgery Division, Magna Graecia University of Catanzaro, 88100 Catanzaro, Italy

**Keywords:** gonial angle, lower third molar, third molar impaction, J&D classification, facial typology

## Abstract

Lower third molars (M3M) are the most frequent impacted teeth. The aim of this study was to evaluate the correlation between M3M position and gonial angle. A retrospective cross-sectional study was conducted. The study population included patients with unilateral or bilateral M3M and underwent Cone Beam Computed Tomography. A morphometric analysis of the mandible was performed after three-dimensional reconstruction, recording gonial angle (GA), ramus high, ramus width, ramus divergency, and retromolar space. GA was the primary predictor variable. The primary outcome variable was the position of M3M analyzed in sagittal, axial, and coronal planes. Descriptive, bivariate, and multiple regression statistics were performed (*p* < 0.05). Study sample included 172 patients (mean age: 26.3 ± 4.6 years); 266 M3Ms were analyzed. The average GA was 122.6° ± 4.8°. A reduced GA value was significantly associated with a deeply impacted M3M in the ramus. With a progressive decrease of GA, M3M assumed a more horizontal position closer to the mandibular canal (*p* < 0.05). A lower GA showed a reduced retromolar space with more complex impacted M3M (*p* < 0.05). The results confirm a statistically significant correlation between GA and the position of M3M. Higher incidence of impacted M3M was related to a reduction of the GA value.

## 1. Introduction

The lower third molars (M3M) are the most frequent impacted teeth with indication to surgical removal [1,2]. The surgical treatment of the M3M always represents a challenge for oral surgeons, considering difficulties and possible risks of this procedure [3]. Surgical removal of the lower third molars is strictly related to anatomical factors and operative abilities of the surgeon [4]. Several radiologic classifications aimed to determine the surgical difficulty of the M3M removal [5]. Winter and Pell-Gregory implemented two different classification models, assessing the position of the M3M on panoramic radiograph [6,7]. Despite these classification systems being easy and intuitive, they are missing in providing some fundamental information to the surgeon because they are based on two-dimensional (2D) exams [8,9]. For this reason, a significative update was introduced by Stacchi and colleagues, estimating the difficulty of the third molar surgery after analyzing Cone Beam computed tomography (CBCT) scans in the three different planes [10]. To date, a three-dimensional (3D) radiological volume rendering of the M3M is often required, giving the practitioner a more detailed view of the anatomical features than 2D imaging techniques [11,12,13]. Several etiological factors contribute to M3M impaction, but the craniofacial development certainly represents the main aspect [14,15]. In 1956, characteristic signs of mandibular morphology in relation to the impacted M3M were identified [16]. The lower third molar impaction was mainly associated to a reduced mandibular growth in length and a vertical direction of mandibular condyle related to an insufficient resorption of anterior border of the ramus [16]. Patients with skeletal class II showed high probability of M3M impaction because they recorded a smaller mandible with more acute gonial angle [17]. In the last decades, many studies aimed to evaluate the correlation between the facial typology and the mandibular third molar position [15,17,18,19,20]. In particular, the facial typology was defined as a direct consequence of the mandibular growth pattern, and the gonial angle well represents its clinical expression [21,22]. The growth rotation of the mandible could distinguish dolichofacial, mesofacial, and brachyfacial typology, assessing the specific skeletal characteristics in vertical and horizontal planes [22]. Dolichofacial typology records an excess vertical growth pattern, a clockwise rotation of the mandible, and clinical features of a long face with weak muscles and lip incompetence [21,23]. Mesofacial typology shows a harmonic growth of maxillary and mandibular bone both in vertical and horizontal plane [24]. On the contrary, brachyfacial typology is characterized by a decreased vertical growth pattern and a counter-clockwise rotation of the mandible, defining the clinical aspect of a short and squared face with strong muscles [21].

To date, the correlation between mandibular morphology and the M3M impaction has been studied using 2D radiographs [16,17,18,19]. There is a knowledge gap on 3D assessment which would allow the position of the M3M to be more accurately related to the surrounding anatomical structures.

The purpose of this study was to analyze the correlation between the gonial angle and the lower third molar position using CBCT scans and 3D reconstruction of the mandible. The authors hypothesized that the mandibular morphology could not influence the development of M3M. The specific aim of the study was to correlate the gonial angle to the position of M3M in relation to the (1) second molar (M2M), (2) mandibular ramus, (3) alveolar crest, (4) inferior alveolar nerve (IAN), (5) buccal or lingual wall, and (6) spatial position.

## 2. Materials and Methods

### 2.1. Study Design

The study was designed as a retrospective single-center cross-sectional study. The medical protocol and ethics followed the Declaration of Helsinki. The regional ethical review board (reference for the Magna Graecia University of Catanzaro, Catanzaro, Italy) approved the study (n. 172/2020).

### 2.2. Study Sample

The study sample included CBCT collected up to November 2020 at the Oral and Maxillofacial Surgery Department of Magna Graecia University of Catanzaro. Eight hundred and fifty-six CBCTs were screened, but only 172 patients were eligible for the analysis. To use radiologic data for scientific analysis, a specific informed consent form was signed by all patients. Inclusion criteria were the following: (1) patients aged 18 to 32 years; (2) no previous orthodontic treatment; (3) presence of unilateral or bilateral M3M (3.8 and/or 4.8); (4) extension of the CBCT scans from the nasal bones to the chin; (5) high resolution images without metal artifacts and/or movement; (6) presence of central incisors, first and second molars without altered angulation in both dental arches; (7) dental arches in centric occlusion during scans acquisition; (8) completed root apexification (stage H) [25]. Patients with history of mandibular trauma, facial malformations, craniofacial syndromes, systemic diseases, osteometabolic disorders, parathyroid diseases, or incomplete radiological exams were excluded.

### 2.3. Data Collection Method

At baseline, data collection was based on anamnestic and demographic data, clinical evaluation, and radiographic tools.

The analysis of Digital Imaging and Communications in Medicine (DICOM) files allowed to identify and classify lower third molars (M3M) according to Juodzbalys and Daugela [26] (Supplementary Table S1). CBCT images were obtained using Vatech PaX-Reve3D (FOV 15 × 15; 50–100 kVp; 1–22 mA; 200 µ) (Vatech, Fort Lee, NJ, USA). According to the classification, anatomical and radiological criteria allowed to evaluate the M3M spatial position (S) and the M3M position in relation to the lower second molar (M2M) (M), mandibular ramus (R), alveolar crest (A), inferior alveolar nerve (IAN) (C), and buccal or lingual wall (B). For each parameter, score ranged among 0 and 3, whereas total score ranged between 0 and 18. Two investigators (AA and FD) separately conducted the lower third molar classification. Any disagreements between the two authors were discussed and judged by a third expert author (AG).

**Figure 1:**
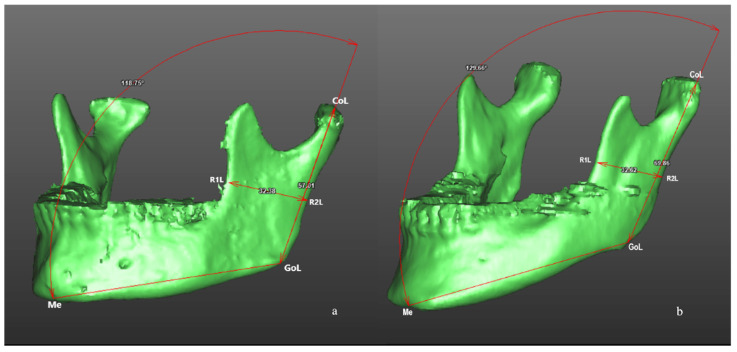
Morphometric analysis of the mandible after three-dimensional volumetric reconstruction of CBCT scans in a patient of L-GA group (**a**) and in a patient of H-GA group (**b**). Linear measurements included mandibular ramus high (Co-Go) and mandibular ramus width (R1-R2). Angular measurement included gonial angle (Condilion-Gonion-Menton; Co-Go-Me).

**Figure 2:**
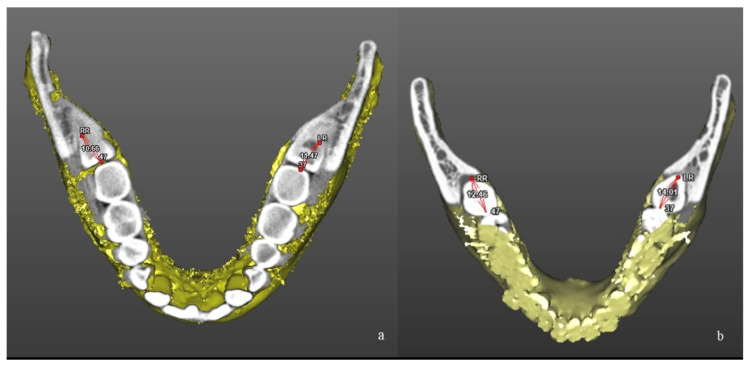
Linear measurement of the retromolar space after three-dimensional reconstruction of CBCT scans in a patient of L-GA group (**a**) and in a patient of H-GA group (**b**). The retromolar space was calculated as the distance between the distal surface of the second molar and the anterior border of mandibular ramus (3.7—Left Ramus; 3.7-LR; 4.7—Right Ramus; 4.7—RR).

The morphometric analysis of the mandible was performed processing DICOM files through a dedicated software (SimPlant**^®^** O&O 2.5, O&O Software, Materialise Dental n.v., Technologielaan 15 3001 Leuven, Belgium) to obtain the tomographic segmentation and the volumetric three-dimensional reconstruction of the mandible. A calibrated researcher (FA) recorded the following measurements: (1) gonial angle (angle defined by three points: Condilion-Gonion-Menton; Co-Go-Me) (Figure 1); (2) mandibular ramus high (linear measurement defined by two points: Co-Go); (3) mandibular ramus width (linear measurement defined by two points: R1-R2); (4) distance between the most distal point of the crown of the M2M and the anterior border of mandibular ramus, defined as retromolar space (M2M—ramus; 7-R) (Figure 2); (5) mandibular ramus divergency, calculating the angle between mandibular ramus plane (Co-Go-R2) and sagittal plane.

### 2.4. Study Variables and Outcomes

The primary predictor variable was the gonial angle, defined between the tangent line to the posterior border of mandibular ramus and the tangent line to the inferior border of mandibular body (Co-Go-Me). Two groups were distinguished: high- and low-gonial angle (H-GA; L-GA).

The primary outcome variable was the position of the M3M analyzed in sagittal, axial, and coronal planes, according to Juodzbalys and Daugela (JD) (Table S1 Supplementary Materials) [26].

Other study variables were recorded: patients’ age and gender, mandibular ramus high, mandibular ramus width, and distance between the M2M and the anterior border of mandibular ramus.

### 2.5. Statistical Analysis

Sample size calculation was performed, setting input parameters (effect size f2 = 0.05, significant level α = 0.05, power 95%, number of predictors = 1). A total of 262 cases were necessary for this study. Continuous variables were reported using mean and standard deviation. Categorical data were recorded as frequencies and percentages. Descriptive, bivariate, and multiple regression statistics were performed. Bivariate analysis included Student’s t-test for the comparison of quantitative continuous variables and Chi-square test for categorical variables. Coefficient (β), and *p*-value were recorded. A *p*-value < 0.05 was considered statistically significant. The inter-rater agreement between the two investigators (AA and FD) was calculated using the Cohen’s kappa coefficient (κ) [27]. Statistical analysis was performed using the software STATA (STATA version 11, StataCorp, College Station, TX, USA).

## 3. Results

The study sample included 172 patients (66 women and 106 men) with 266 lower third molars (M3M). All patients’ data are shown in Table 1. The gonial angle showed a mean value of 122.6° ± 4.8° and it represented the cut-off for the high- and the low-gonial angle groups (H-GA; L-GA). The H-GA group included 127 lower third molars (47.7%), with a mean gonial angle of 126.8° ± 2.8°. The L-GA group included 139 lower third molars (52.3%), with a mean gonial angle of 118.8° ± 2.5°. The inter-rater agreement between the two investigators (AA and FD) was κ = 0.93.

Table 2 summarized the bivariate comparison between the predictor variable and other variables, showing a statistically significant difference between the H-GA and the L-GA groups. Ramus length and ramus width were significantly greater in the L-GA group than the H-GA group (*p* < 0.0001).

Table 3 summarized the bivariate comparison between the predictor variable and the primary outcome variable, showing a statistically significant difference between the HGA and the LGA groups in terms of M, R, A, B, S, and JD score (*p* < 0.05).

Table 4 summarized the multivariate analysis, in relation to the primary outcome variable.

- Position of M3M in relation to M2M—M

A significant correlation was recorded between M and patients’ age (*β* = −0.03; CI: −0.05, −0.001; *p* = 0.04): M score increased with the decreasing age. The gonial angle was significantly associated with M (*β* = −0.03; CI: −0.05, 0.001; *p* = 0.05): M score increased with decreasing gonial angle. The distance between the mandibular ramus and the M2M (7-R) showed a significant correlation with M (*β* = −0.16; CI: −0.04, −0.1; *p* < 0.0001): M score increased with decreasing 7-R.

- Position of M3M in relation to mandibular ramus—R

A significant correlation was recorded between R and the gonial angle (β = −0.05; CI: −0.07, −0.02; *p* = 0.001): R score increased with the decreasing of the gonial angle. The distance between the mandibular ramus and the M2M (7-R) was significantly associated with R (β = −0.2; CI: −0.2, −0.1; *p* < 0.0001): R score increased with decreasing 7-R.

- Position of M3M in relation to alveolar crest—A

**Figure 3:**
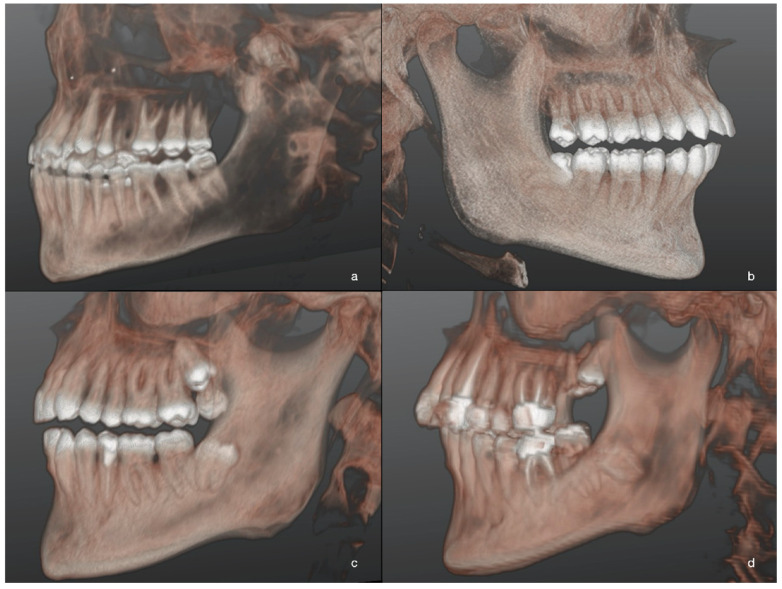
Three-dimensional images of M3M after 3D reconstructions of CBCT scans in patients of L-GA group. Different levels of M3M position in relation to the adjacent alveolar crest were reported: the most superficial M3M, completely erupted (**a**); partially impacted M3M in horizontal position (**b**); completely impacted M3M in the ramus with distoangular position (**c**); the deepest impacted M3M with roots closer to mandibular canal (**d**).

**Figure 4:**
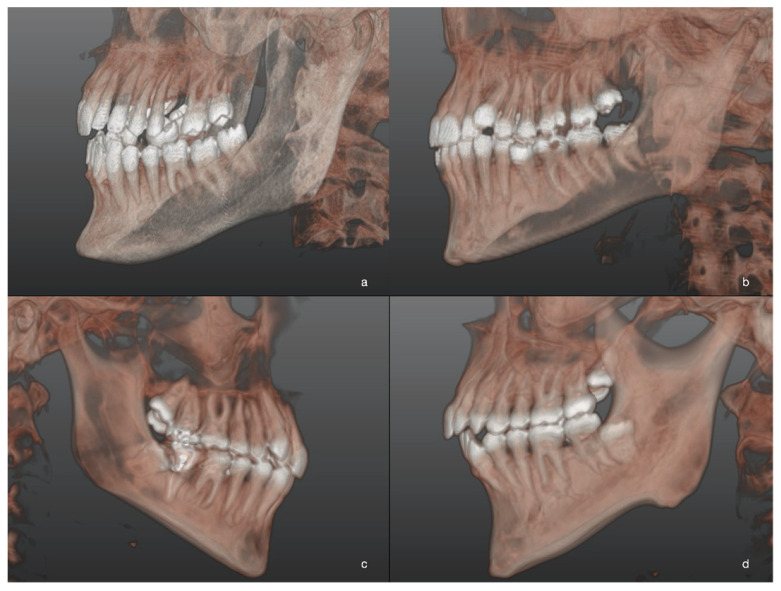
Three-dimensional images of M3M after 3D reconstructions of CBCT scans in patients of L-GA group. Different levels of M3M position in relation to the adjacent alveolar crest were reported: the most superficial M3M, completely erupted (**a**); partially impacted M3M in horizontal position (**b**); completely impacted M3M in the ramus with distoangular position (**c**); the deepest impacted M3M with roots closer to mandibular canal (**d**).

A significant correlation was recorded between A and the gonial angle (β = −0.; CI: −0.1, −0.01; *p* = 0.004): A score increased with the decreasing of the gonial angle (Figure 3; Figure 4). The distance between the mandibular ramus and the M2M (7-R) was significantly associated with A (β = −0.; CI: −0.2, −0.1; *p* < 0.0001): A score increased with decreasing 7-R.

- Position of M3M in relation to inferior alveolar nerve—C

The distance between the mandibular ramus and the M2M (7-R) was significantly associated with C (*β* = −0.1; CI: −0.1, −0.01; *p* = 0.02): C score increased with decreasing 7-R.

- Position of M3M in relation to buccal or lingual wall—B

A significant correlation was recorded between B and gender (*β* = −0.3; CI: −0.6, −0.07; *p* = 0.01): B score increased in male patients. The gonial angle was significantly associated with B (*β* = −0.03; CI: −0.05, −0.01; *p* = 0.0007): B score increased with the decreasing gonial angle. A significant correlation was found between B and ramus width (*β* = 0.04; CI: 0.002, 0.08; *p* = 0.04): B score increased with increasing ramus width.

- Spatial position of M3M—S

A significant correlation was recorded between S and gender (*β* = −0.4; CI: −0.7, −0.1; *p* = 0.01): S score increased in male patients. The distance between the mandibular ramus and the M2M (7-R) showed a significant correlation with S (*β* = −0.11; CI: −0.2, −0.06; *p* < 0.0001): S score increased with decreasing 7-R. A significant correlation was determined between S and rams high (*β* = 0.03; CI: 0.001, 0.05; *p* = 0.04): S increased with increasing mandibular rams high.

- JD score—JD

A significant correlation was recorded between the JD and the gonial angle (*β* = −0.2; CI: −0.3, −0.06; *p* = 0.004): the JD score increased with the decreasing gonial angle. The distance between the mandibular ramus and the M2M (7-R) showed a significant correlation with JD (*β* = −0.7; CI: −0.9, −0.4; *p* < 0.0001): the JD score increased with decreasing 7-R.

## 4. Discussion

This retrospective study aimed to perform a morphometric analysis of the mandible after a 3D reconstruction of CBCT scans in order to evaluate the correlation between mandibular morphology and the lower third molar position. Facial typology and mandibular growth could influence the eruptive pattern and development of the M3M [16,17,18,19,20]. To our knowledge, this is the first three-dimensional study aiming to evaluate the M3M position and correlating it to the facial typology. Specifically, in this study the position of the M3M was three-dimensionally analyzed in relation to the M2M, mandibular ramus, alveolar crest, IAN, buccal or lingual wall, and spatial position, also defining the level of difficulty for a surgical treatment.

The results confirmed our hypothesis, showing a statistically significant difference in the M3M position between mandibles with high- and low-gonial angles. Characteristic features of the H-GA and the L-GA groups were determined, suggesting a significant difference in their mandibular morphologies. Mandibular ramus length and ramus width were significantly greater in the L-GA group than the H-GA group. In this analysis, the gonial angle represented the main measurement of mandibular morphology. It was calculated on volumetric elaboration of CBCT scans using a dedicated software for the 3D cephalometric examination. As reported in literature, the gonial angle is closely related to the growth pattern and facial typology [18,28,29]. According to D’Antò (et al.), the GA could help to define the mandibular rotational pattern with more accurate information than the angle between anterior cranial base and mandibular plane that is influenced by the anterior cranial base inclination [30,31]. Dolichofacial people showed a clockwise mandibular growth pattern and an increased anterior facial height, with high values of the GA; on the contrary, in brachyfacial individuals a counter-clockwise mandibular rotation, a decreased anterior facial height, and a lower GA were recorded [18]. As highlighted by our results, a decreased gonial angle with a consequent counter-clockwise rotation of the mandible is significantly associated with an altered M3M impaction. Specifically, the analysis of the mesiodistal position of the M3M showed a closer relation with the M2M and mandibular ramus in L-GA group, because a limited retromolar space and a complex eruptive pattern exist. In patients with a small gonial angle, the M3M crown was often directed from the middle to the apical third of the M2M root, while its root development occurred often in mandibular ramus. Despite patients’ age not differing between H-GA and L-GA, a reduced resorption of the mandibular ramus cortex could not predispose the M3M to perform the physiologic eruptive curve in the L-GA group. Apico-coronal position analysis in relation to the alveolar crest revealed that patients with a low gonial angle and a counter-clockwise mandibular rotation frequently showed a complete bone impaction of the M3M. In these patients, the lower third molar should design a concave and more angled eruptive path with severe impaired cortical resorption. Evaluating the buccolingual position, our sample showed that the M3M was closer to lingual wall in the L-GA mandibles, in which a major ramus divergency was also determined. Mandibular ramus divergency is an innovative measurement that could be calculated thanks to 3D methodologies of this study. It may interfere with the M3M eruption because in the L-GA group, the greater the mandibular ramus divergency, the greater the convergent lingual development of the crown and the divergent buccal development of the roots, following the mandibular anatomical structure. All detected items also revealed that the M3M position was directly dependent on the space between the M2M and the anterior mandibular ramus border. Therefore, the analysis of spatial position indicated that a reduced retromolar space was significantly associated with a horizontal, deeply impacted lower third molar. A small mandibular plane angle could influence the development and position of the M3M because its eruption pattern is hampered by a reduced retromolar space and a significant ramus width.

Because D’Antò and colleagues found a strong positive association between the GA and the angle between anterior cranial base and mandibular plane, we can conclude that the counter-clockwise mandibular growth could predispose to the deepest position of the M3M that should take a more complex eruptive path in mandibles with an acute gonial angle [30]. In the L-GA group, the disadvantages of a hypodivergent growth pattern could interfere with the angulation of the M3M impaction, predisposing to a complete or partial M3M impaction. The evaluation of the M3M position with a three-dimensional approach allowed also to summarize fundamental information for oral surgeon. Although 2D radiograph represents the initial screening test for third molar surgery, it gives only limited data in terms of roots morphology, buccal-lingual position of M3M, relationship with the mandibular canal, and alteration of IAN pathway. Pre-surgical evaluation of CBCT can guarantee a detailed evaluation of M3M and surrounding structures with a certain accuracy that should avoid or limit intra-operative or post-surgical complications [32,33,34]. In particular, the calculation of the JD score could be useful to define the level of difficulty of the surgical treatment. Our sample showed higher values of the JD score in the L-GA group, indicating a more complex intervention of the M3M removal in hypodivergent patients with a significantly reduced retromolar space. The interpretation of our results could suggest that hypodivergent patients may benefit from an early extraction of M3M. Germectomy could avoid possible worsening of clinical conditions after tooth development and complete apexification [35]. According to the third molar classification, the difficulty of the surgical removal can range from moderate to complicated when at least one parameter is equal to score 2 or 3, respectively, increasing the risk during surgery. Therefore, early treatment could be indicated for patients of L-GA group.

Mandibular morphology has a central role in the overall facial growth and the gonial angle characterizes, specifically the growth pattern of the mandible, influencing its vertical development [5,28,36,37]. The direction of mandibular growth is related with a hyperdivergent or hypodivergent facial typology, showing high or low gonial angle, respectively [30,36,38]. As described in literature, the different clinical characteristics of these two mandibular patterns of growth concern also the position and the eruptive path of the M3M [16,17,18,19,20]. Björk and colleagues conducted detailed studies on facial growth development, concluding that the mandibular morphology could influence the lower third molar eruption [16]. A decreased mandibular body length and a limited resorption of the anterior ramus border are closely linked to the risk of the impacted M3M [16]. Several studies investigated the specific correlation between the gonial angle and the M3M position on panoramic radiographs, aiming to define the most favorable mandibular morphology for lower third molar eruption (Table 5) [18,19,20,29]. However, no consistent data is recorded.

As reported by Richardson, a counter-clockwise rotation of the mandible with a reduced gonial angle increases the probability of the M3M impaction [17]. In accordance with our results, Gümrükçü and colleagues in a group of 601 patients, aged between 18 and 30 years, determined that an increased gonial angle may favor the third molar eruption because the upward mandibular rotation could limit the resorption of the anterior border of the ramus, reducing the third molar eruption space and predisposing to the M3M impaction [19]. Contrasting results were recorded by Hattab and Alhaija, Demirel and Akbulut analyzing a group of in-growing patients and adult subjects, respectively [18,39]. They used panoramic radiographs to calculate the GA, and no significant association between facial typology and mandibular third molar impaction was found [18,39]. Other studies reported a greater prevalence of impacted M3M in dolichofacial subjects, because brachyfacial typology allows a greater horizontal growth, favoring resorption of the anterior border of the mandibular ramus [20,29,39,40,41,42,43,44,45]. However, because Abu Alhaija and colleagues recorded higher incidence of the M3M impaction in class III malocclusion, they did not agree that the potential growth could be the only fundamental factor for the M3M eruption [42]. Although Al-Gunaid reported an increased risk of the M3M impaction in larger GA patients, in their 2D x-rays analysis, the status of M3M was distinguished only between impaction and normal eruption, without an accurate difference between complete or partial impaction [40]. As described by Legovic, the angulation of the M3M has to be evaluated because the greater the mandibular counter-clockwise rotation, the higher the M3M angulation, in accordance with our results [46]. Moreover, the mandibular space between the distal surface of the M2M and the anterior ramus border is defined as one of the most important elements for a correct eruptive pattern of the M3M [40,41,47]. Ventä and colleagues reported that a space of 16.5 mm corresponds to a probability of the M3M eruption equal to 100% [48]. Uthman and colleagues declared that this retromolar space has to be greater than 11–12 mm. According to the results of this three-dimensional study, undoubted evidence exists that a reduced retromolar space is significantly associated to the M3M impaction, considering also that the ratio between this space and the mesiodistal dimension of the M3M crown must be >1 [49].

The major strength of this retrospective study is the analysis of CBCT scans with 3D reconstruction of skeletal structures and the M3M classification in the three space planes. However, the study design as a single-center study is limited. The southern Italian population frequently includes people with counter-clockwise mandibular rotation that could influence the cut-off value for the H-GA and the L-GA groups [30].

## 5. Conclusions

Our results highlighted a statistically significant correlation between the position of the M3M and the mandibular growth pattern. A lower GA was significantly associated with higher incidence of impacted M3M. In particular, the morphometric analysis showed that a lower gonial angle was related with a reduced retromolar space, favoring the development of the M3M in a complete bone impaction, with a horizontal position and closer to IAN. In addition, the results of this three-dimensional study can help define the prognosis of the M3M in relation to mandibular growth, providing the surgeon with significant details on the timing and planning of surgical removal of the M3M. Based on the findings of this paper, future prospective studies with clinical data should aim to clarify whether germectomy could be a beneficial approach in patients with specific facial features.

## Figures and Tables

**Table 1:** Descriptive statistics of the study sample.

	Study Sample *n* (%)
Patients	172 (100)
M3M	266 (100)
3.8	128 (48.1)
4.8	138 (51.9)
Gonial angle (°)	122.6 ± 4.8
H-GA	126.8 ± 2.8
L-GA	118.8 ± 2.5
Age (years)	26.3 ± 4.6
Sex	
Male	169 (63.5)
Ramus length (mm)	67.8 ± 6.2
Ramus width (mm)	31.2 ± 3.0
Ramus divergency (°)	65.5 ± 22.9
Retromolar space (mm)	11.5 ± 2.1
JD classification	
M	
0	148 (55.6)
1	53 (20)
2	50 (18.8)
3	15 (5.6)
R	
0	145 (54.5)
1	59 (22.2)
2	39 (14.7)
3	23 (8.6)
A	
0	132 (49.7)
1	36 (13.5)
2	54 (20.3)
3	44 (16.5)
C	
0	90 (33.8)
1	108 (40.6)
2	45 (16.9)
3	23 (8.7)
B	
0	4 (1.5)
1	75 (28.2)
2	105 (39.5)
3	82 (30.8)
S	
0	141 (53)
1	86 (32.3)
2	14 (5.3)
3	25 (9.4)
JD score	
0–6	154 (57.9)
7–12	83 (31.2)
13–18	29 (10.9)

M3M = lower third molar; H-GA = High gonial angle; L-GA = Low gonial angle; JD = Juodzbalys and Daugela classification; M = second molar; R = mandibular ramus; A = alveolar crest; C = inferior alveolar nerve (IAN); B = buccal or lingual wall; S = spatial position.

**Table 2:** Bivariate comparisons between the primary predictor variable (gonial angle) and the other variables.

	High Gonial Angle	Low Gonial Angle	*p*-Value
Study sample			
Patients	86	86	Not applicable
Lower third molar	127 (47.7%)	139 (52.3%)	Not applicable
Age (years)	26.1 ± 4.5	26.4 ± 4.7	0.2
Sex			0.03 *
Male	72 (56.7%)	97 (69.8%)	
Ramus length (mm)	65.6 ± 6	69.8 ± 5.7	<0.001 *
Ramus width (mm)	30.1 ± 2.8	32.1 ± 3	<0.001 *
Ramus divergency (°)	63.7 ± 23.3	67 ± 22.5	0.06
Retromolar space (mm)	11.6 ± 2.1	11.4 ± 2.2	0.18

* Statistically significant *p*-values (*p* < 0.05).

**Table 3:** Bivariate comparisons between the primary predictor variable (gonial angle) and the primary outcome variable (third molar classification).

	High Gonial Angle *n* (%)	Low Gonial Angle *n* (%)	*p*-Value
Juodzbalys and Daugela classification			
Relation to the second molar (M)			0.009 *
0	80 (62.9)	68 (48.9)	
1	26 (20.5)	27 (19.4)	
2	19 (15)	31 (22.3)	
3	2 (1.6)	13 (9.4)	
Relation to the mandibular ramus (R)			0.0003 *
0	81 (63.8)	64 (46.1)	
1	31 (24.4)	28 (20.1)	
2	11 (8.7)	28 (20.1)	
3	4 (3.1)	19 (13.7)	
Relation to the adjacent alveolar crest (A)			0.01 *
0	72 (56.7)	60 (43.2)	
1	20 (15.8)	16 (11.5)	
2	23 (18.1)	31 (22.3)	
3	12 (9.4)	32 (23)	
Relation to the mandibular canal (C)			0.2
0	45 (35.4)	45 (32.4)	
1	56 (44.1)	52 (37.4)	
2	15 (11.8)	30 (21.6)	
3	11 (8.7)	12 (8.6)	
Relation to mandibular lingual and buccal walls (B)			0.001 *
0	3 (2.4)	1 (0.7)	
1	48 (37.8)	27 (19.4)	
2	49 (38.6)	56 (40.3)	
3	27 (21.3)	55 (39.6)	
Spatial position (S)			0.04 *
0	72 (56.7)	69 (49.6)	
1	43 (33.9)	43 (31)	
2	7 (5.5)	7 (5)	
3	5 (3.9)	20 (14.4)	
Juodzbalys and Daugela score (JD)			0.001 *
0–6	86 (67.7)	68 (49)	
7–12	35 (27.6)	48 (34.5)	
13–18	6 (4.7)	23 (16.5)	

* Statistically significant *p*-values (*p* < 0.05).

**Table 4:** Multiple linear regression model for the primary outcome variables (M, R, A, C, B, S, JD).

	Coefficient *β*	*p*-Value
(Constant)	M: 5.3	M: 0.02 *
	R: 8.4	R: 0.0002 *
	A: <0.001	A: 0.001 *
	C: 4.7	C: 0.04 *
	B: 4.9	B: 0.01 *
	S: 3.8	S: 0.08
	JD: 36.7	JD: 0.0005 *
Sex	M: −0.14	M: 0.3
	R: −0.3	R: 0.09
	A: <0.0001	A: 0.2
	C: 0.05	C: 0.8
	B: −0.3	B: 0.01 *
	S: −0.4	S: 0.008 *
	JD: −1.3	JD: 0.07
Age	M: −0.03	M: 0.04 *
	R: −0.02	R: 0.2
	A: <0.0001	A: 0.055
	C: −0.01	C: 0.4
	B: −0.01	B: 0.3
	S: −0.01	S: 0.7
	JD: −0.1	JD: 0.09
Lower third molar (3.8 or 4.8)	M: −0.1	M: 0.3
	R: −0.04	R: 0.7
	A: <0.0001	A: 0.5
	C: −0.1	C: 0.4
	B: −0.06	B: 0.5
	S: −0.03	S: 0.8
	JD: −0.5	JD: 0.4
Gonial angle	M: −0.02	M: 0.05 *
	R: −0.04	R: 0.001 *
	A: <0.0001	A: 0.004 *
	C: −0.01	C: 0.5
	B: −0.03	B: 0.007*
	S: −0.02	S: 0.1
	JD: −0.2	JD: 0.004 *
Ramus high	M: 0.01	M: 0.4
	R: 0.0005	R: 0.9
	A: <0.0001	A: 0.9
	C: −0.01	C: 0.5
	B: 0.004	B: 0.7
	S: 0.03	S: 0.04 *
	JD: 0.03	JD: 0.6
Ramus width	M: 0.006	M: 0.8
	R: 0.005	R: 0.8
	A: <0.0001	A: 0.9
	C: −0.04	C: 0.1
	B: 0.04	B: 0.04 *
	S: −0.03	S: 0.3
	JD: −0.01	JD: 0.9
Retromolar space	M: −0.2	M: <0.0001 *
	R: −0.2	R: <0.0001 *
	A: <0.0001	A: <0.0001 *
	C: −0.07	C: 0.02 *
	B: 0.0002	B: 0.9
	S: −0.1	S: <0.0001 *
	JD: −0.7	JD: <0.0001 *
Ramus divergency	M: 0.003	M: 0.3
	R: 0.001	R: 0.6
	A: <0.0001	A: 0.2
	C: 0.004	C: 0.1
	B: −0.002	B: 0.4
	S: −0.01	S: 0.8
	JD: 0.01	JD: 0.4
R-squared	M: 0.15	M: <0.0001 *
	R: 0.17	R: <0.0001 *
	A: 0.14	A: <0.0001 *
	C: 0.03	C: 0.05 *
	B: 0.07	B: 0.001 *
	S: 0.1	S: <0.0001 *
	JD: 0.15	JD: <0.0001 *

M3M = lower third molar; JD = Juodzbalys and Daugela classification; M = second molar; R = mandibular ramus; A = alveolar crest; C = inferior alveolar nerve (IAN); B = buccal or lingual wall; S = spatial position; * Statistically significant *p*-values (*p* < 0.05).

**Table 5:** Literature review of the studies investigating the correlation between mandibular morphology and third molar position.

Author, Year	Study Design; Sample (Number of Patients) Radiologic Assessment	Study Outcomes and Conclusions
Hattab et al. 1999 [39]	Retrospective study; 134; orthopantomography	The mean GA was 122.14° in the impacted group and 120.08° in the erupted group. The retromolar space was significantly smaller in the group with impacted M3M and it was also associated with lateral asymmetry of M3M in both groups. The third molar space/crown width ratio was <1 in the impacted group and >1 in the erupted group (*p* < 0.001). The mesiodistal crown width was not significantly different between impacted or erupted group.
Mollaoglu et al., 2002 [47]	Retrospective study; 213; orthopantomography	GA did not differ significantly between the erupted and the impacted groups. It was observed that the M3M mesiodistal angulation was significantly higher in impacted group in which there was a significantly lower retromolar space (*p* < 0.05). The retromolar space/third molar crown width ratio differed significantly between impacted and erupted groups.
Tsai et al., 2005 [41]	Retrospective study; 152; orthopantomography	In male patients, mandibular body length, mandibular ramus width, and first molar width were significantly greater in the impacted group. The retromolar space was significantly lower in the impacted group (*p* < 0.05). In female patients, mandibular ramus high and first molar width were significantly greater in the impacted group. The retromolar space was significantly lower in the impacted group (*p* < 0.05). GA did not show any difference between the groups.
Uthman et al., 2007 [49]	Cohort study; 50; orthopantomography	The retromolar space was significantly lower in marginal-eruption group than in full-eruption group (*p* < 0.01). The retromolar space/M3M width ratio was significantly greater in the full-eruption group (mean ratio >1) than in the marginal-eruption group (mean ratio <1) (*p* < 0.01). GA was not significantly different between the two groups.
Legovic et al., 2008 [46]	Retrospective study; 130; Orthopantomography and lateral radiograph	In male patients, a significant correlation was found between the retromolar space and the vertical position of lower right M3M (*p* < 0.05). In female patients significant correlations were determined: (1) between the retromolar space and the vertical position of M3M (*p* < 0.05); (2) between the retromolar area and M3M inclination (*p* < 0.05); (3) between the retromolar space and spatial relation of M3M (*p* < 0.05). A significant correlation between lower right M3M inclination and anterior facial rotation was observed (*p* < 0.05).
Breik et al., 2008 [20]	Retrospective study; 98; Orthopantomography and lateral radiograph	The mandibular third molar impaction was 58.76%. Brachyfacial patients showed lower incidence of mandibular third molar impaction than dolichofacial patients (*p* < 0.01). No difference was found between mesofacial and dolichofacial patients. Most of the impacted M3Ms were in a horizontal position.
Abu Alhaija et al., 2011 [42]	Retrospective study; 270; Orthopantomography and lateral radiograph	M3M impaction was recorded in 26% of Skeletal Class I, 32% of Skeletal Class II, and 42% of Skeletal Class III. Impacted M3M was significantly associated with reduced retromolar space width, increased angle between M2M and M3M, and decreased M3M angulation in all skeletal patterns (*p* < 0.05). Only in skeletal Class I GA was significantly greater in the impacted group than in the erupted group (*p* < 0.01).
Begtrup et al., 2013 [43]	Retrospective study; 53; Orthopantomography and lateral radiograph	No correlation between jaw angles and M3M eruption was found. A larger distance from the articulare point to the interdentale point is correlated with M3M eruption.
Kanwal et al., 2013 [44]	Descriptive cross-sectional study; 60; Orthopantomography	Frequency of M3M impaction was significantly higher in dolichofacial type (46.67%) than in brachyfacial type (16.67%) (*p* < 0.05). Most female patients showed impacted M3M.
Gupta et al., 2017 [29]	Retrospective study; 150; Orthopantomography and lateral radiograph	Impacted M3Ms were mostly in mesioangular position, (49.3%) followed by distoangular (22.7%) and vertical position (20.2%). M3M impaction occurred in brachyfacial patients (44%), in dolichofacial (49%), and in mesofacial subjects (77%), with a significant difference among the groups (*p* < 0.05).
Tassoker et al., 2019 [45]	Retrospective study; 158; Orthopantomography and lateral radiograph	Brachyfacial patients showed a lower prevalence of third molar impaction, with respect to dolichofacial and mesofacial patients (*p* < 0.05). No correlations were found between skeletal facial type and the angular position of M3M (*p* > 0.05).
Demirel et al., 2019 [18]	Retrospective study; 90; Orthopantomography to evaluate mandibular morphology and CBCT to evaluate M3M	Mean gonial angle value was 121.38° ± 7.64°. The most common position was mesioangular M3M. No significant correlation was found between gonial angle and other variables (age, gender, third molar angulation) (*p* > 0.05). Despite no significant relationship being observed between Pell–Gregory groups and gonial angle values (*p* > 0.05), a significantly higher gonial angle (*p* < 0.05) was found only in the sub-group in which M3M was partially inside the ramus and its occlusal level was below than cervical level of M2M.
Al-Gunaid et al., 2019 [40]	Retrospective study; 240; Orthopantomography	The erupted group showed longer condylar and coronoid length, longer ramus height, wider ramal width, larger retro¬molar space, higher retromolar area to M3M ratio, and larger angle of impaction than impacted group (*p* < 0.05). The impacted group showed significantly larger GA, and larger inclination of lower posterior teeth than the erupted group (*p* < 0.05).
Gümrükçü et al., 2020 [19]	Retrospective study; 601; Orthopantomography and lateral radiograph	The mean value of GA is 123.8 ± 6.9°. According to Pell-Gregory classification, a statistically significant difference was observed in terms of ramus height–gonial angle between Class A and B, between Class B and C (*p* < 0.05). Ramus high was significantly different between Class A and C (*p* < 0.05). According to Winter Classification, gonial angle was significantly higher in class Vertical and significantly lower in class Horizontal (*p* < 0.05).

M3M = third molar; GA = gonial angle; M2M = second molar; CBCT = Cone Beam Computed Tomography.

## Data Availability

The data presented in this study are available on reasonable request from the corresponding author.

## Supplementary Materials

The following are available online at https://www.mdpi.com/article/10.3390/jcm10184057/s1, Table S1: Reworking of JD classification.
