# Supplementary material for: Does Mandibular Gonial Angle Influence the Eruption Pattern of the Lower Third Molar? A Three-Dimensional Study
Source: J Clin Med. 2021 Sep 8;10(18):4057. doi: 10.3390/jcm10184057 (PMC8464884; doi:10.3390/jcm10184057)
Supplement: Supplementary file 1 [file jcm-10-04057-s001.zip › jcm-1363156-supplementary.pdf]

Supplementary Table S1. Reworking of JD classification

	Risk score of potential M3M surgery			
	**Conventional (0)**	**Simple (1)**	**Moderate (2)**	**Complicated (3)**
**Parameters related to the position of M3M**				
Lower second molar (M)	Crown of M3M is at or above the equator of M2M	Crown of M3M is below the equator to the coronal third of M2M	Crown/roots of M3M is/are to the middle third of the root of M2M	Crown/roots of M3M is/are to the apical third of the root of M2M
Mandibular ramus (R)	M3M has sufficient space in dental arch	M3M is partially impacted in the ramus	M3M is completely impacted in the ramus	M3M is completely impacted in the ramus with distoangular or horizontal position
Alveolar crest (A)	Complete eruption of M3M	Partial impaction - the widest part of the dental crown (equator) is above the bone	Partial impaction - The widest part of the dental crown (equator) is below the bone	Complete bone impaction
Mandibular canal (C)	Distance between M3M and mandibular canal ≥ 3 mm	M3M is in contact with mandibular canal (its wall is identifiable)	M3M is in contact with mandibular canal (its wall is not identifiable)	M3M roots surrounding the mandibular canal
Mandibular lingual and buccal walls (B)	Closer to buccal wall	In the middle between lingual and buccal walls	Closer to lingual wall	Closer to lingual wall, with partial or complete bone impaction (A2 or A3)
Spatial position (S)	Vertical position (90°)	Mesioangular position (≤ 60°)	Distoangular position (≥ 120°)	Horizontal (0°) o inverted position (270°)

M3M= lower third molar; H-GA= High gonial angle; L-GA= Low gonial angle; JD= Juodzbalys & Daugela classification
